# Stem/progenitor cell-based transplantation for retinal degeneration: a review of clinical trials

**DOI:** 10.1038/s41419-020-02955-3

**Published:** 2020-09-23

**Authors:** Yiqi Wang, Zhimin Tang, Ping Gu

**Affiliations:** Department of Ophthalmology, Shanghai Ninth People’s Hospital, Shanghai Jiao Tong University School of Medicine, Shanghai Key Laboratory of Orbital Diseases and Ocular Oncology, Shanghai, 200011 P.R. China

**Keywords:** Self-renewal, Stem-cell differentiation

## Abstract

Retinal degeneration (RD) is one of the dominant causes of irreversible vision impairment and blindness worldwide. However, the current effective therapeutics for RD in the ophthalmologic clinic are unclear and controversial. In recent years, extensively investigated stem/progenitor cells—including retinal progenitor cells (RPCs), embryonic stem cells (ESCs), induced pluripotent stem cells (iPSCs) and mesenchymal stromal cells (MSCs)—with proliferation and multidirectional differentiation potential have presented opportunities to revolutionise the ultimate clinical management of RD. Herein, we provide a comprehensive overview on the progression of clinical trials for RD treatment using four types of stem/progenitor cell-based transplantation to replace degenerative retinal cells and/or to supplement trophic factors from the aspects of safety, effectiveness and their respective advantages and disadvantages. In addition, we also discuss the emerging role of stem cells in the secretion of multifunctional nanoscale exosomes by which stem cells could be further exploited as a potential RD therapy. This review will facilitate the understanding of scientists and clinicians of the enormous promise of stem/progenitor cell-based transplantation for RD treatment, and provide incentive for superior employment of such strategies that may be suitable for treatment of other diseases, such as stroke and ischaemia–reperfusion injury.

## Facts

- Retinal degeneration (RD) is one of the dominant causes of irreversible vision impairment and blindness worldwide.
- Stem/progenitor cell-based transplantation has been extensively investigated for RD therapy.
- Stem/progenitor cells—mainly including retinal progenitor cells (RPCs), embryonic stem cells (ESCs), induced pluripotent stem cells (iPSCs) and mesenchymal stromal cells (MSCs)—exert effects on sight restoration by replacing and/or rescuing degenerative retinal cells and by secretion of multifunctional nanoscale exosomes.

## Open questions

- What are the exact proliferation and differentiation mechanisms of stem/progenitor cells?
- How can the potential tumorigenicity of stem/progenitor cells and immune rejection caused by exogeneous transplantation strategies be overcome?
- How can rapid clearance of nanovesicle exosomes from tissues or organs be avoided?

## Introduction

Retinal degeneration (RD) is a group of diseases causing blindness via progressive visual loss in humans^1^, and includes age-related macular degeneration (AMD)^2^, diabetic retinopathy (DR)^3^, Stargardt’s disease (STGD)^4^ and retinitis pigmentosa (RP)^5^. In particular, AMD is one of the most common ocular diseases clinically, has a global prevalence of 8.7% with an age of onset varying from 45 to 85 years^6^ and is estimated to affect ~196 million individuals in western countries by 2020 and 288 million by 2040^7^. In addition to AMD, DR is also highly prevalent^8^, accounting for ~8.2% of the global adult population with vision loss^9^. Different from AMD and DR, STGD affects approximately one in 10,000 births^10^, and the total prevalence of different forms of RP varies from one in 2500 to 7000 persons^11^. The human retina is a delicate and elaborate thin sheet composed of ten sublayers^12^, including (1) the inner limiting membrane (ILM), (2) nerve fibre layer (NFL), (3) ganglion cell layer (GCL), (4) inner plexiform layer (IPL), (5) inner nuclear layer (INL), (6) outer plexiform layer (OPL), (7) outer nuclear layer (ONL), (8) outer limiting membrane (OLM), (9) photoreceptor layer (PL) and (10) retinal pigmented epithelium (RPE) monolayer. The photoreceptors play an indispensable role in sensing light signals and visual cues through converting exogenous cues into bioelectrical signals^13^, whereas the RPE cells as a layer of pigment cells transport ions, water and metabolic end products from the subretinal space to the blood, and provide ingested nutrients from the blood to photoreceptors^14^. Although there are differences in pathological progression of various RD diseases, it is currently considered that RPE and/or photoreceptor dysfunction is the predominate common pathogenesis of RD^15^, especially when RPE atrophy causes secondary choriocapillaris loss and photoreceptor degeneration, and subsequently results in the detrimental circulatory effects in the dysfunctional RPE and degenerative photoreceptors^16^.

**Scheme 1:**
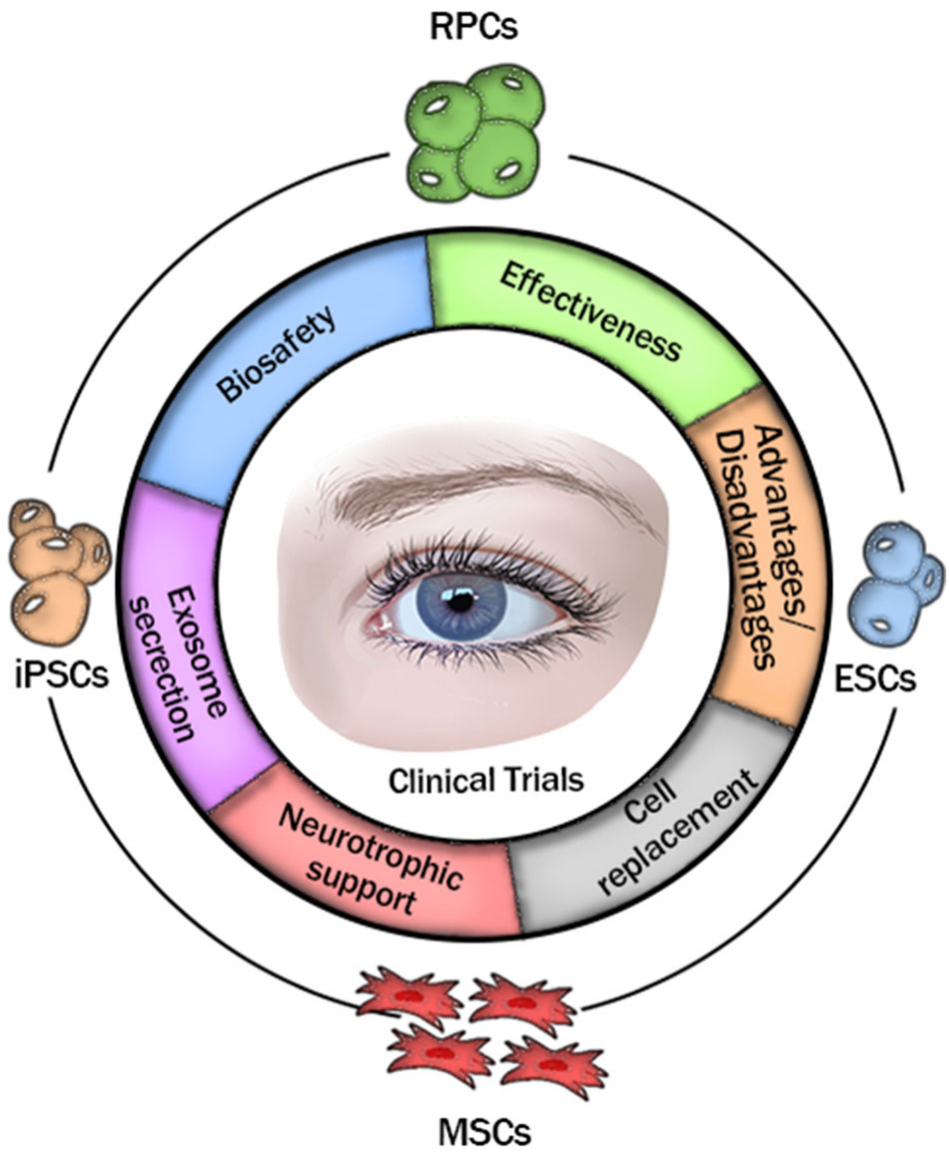
Stem/progenitor cell-based transplantation for retinal degeneration in clinical trials. The safety, effectiveness and advantages and disadvantages of four stem/progenitor cells, i.e., RPCs, ESCs, induced pluripotent stem cells (iPSCs) and MSCs upon transplantation for RD therapy during clinical trials have been discussed, in which they play a critical role in sight restoration through cell replacement, neurotrophic support and the secretion of multifunctional nanoscale exosomes.

Given the high morbidity of RD threatening all age group burdens of the world, it is urgent to provide effective therapeutic strategies for RD management. Currently, RD patients are routinely recommended to receive medical management, including antioxidants^17^, anti-vascular endothelial growth factor (anti-VEGF) agents^18^, neuroprotective strategies^19^, laser or surgery therapy^20^. Among them, ophthalmologic antioxidant cocktails (e.g., vitamins^21^, lutein and zeaxanthin^22^) have been applied to protect retinal cells from oxidative damage, yet the therapeutic outcomes are unsatisfactory due to the unfriendly schedule and underlying biosafety concerns (such as potential risks of skin rashes^23^, haemorrhagic stroke^24^ and lung cancer in cigarette smokers^25^). Injection of anti-VEGF agents, including ranibizumab^26^, aflibercept^27^ and bevacizumab^28^, which bind to the VEGF receptors to block VEGF, is mainly used to treat wet AMD^29^ via inhibition of choroidal neovascularisation^30^. However, adverse reactions of the eyes (such as endophthalmitis, uveitis, retina split holes and vitreous haemorrhage) and systemic adverse reactions (such as hypertension, myocardial infarction and stroke) caused by frequent intravitreal injections and the high cost of treatment lead to poor patient compliance and compromised effectiveness^31^. Neuroprotective interventions are generally divided into two categories^19^—drugs including steroids^32^, dopamine-related therapies^33^ and neurotrophic factors^34^, and rehabilitative methods including physical exercise and electrical stimulation^35,36^; they have been widely used in numerous fundamental studies to slow degenerative progress in the retina by protecting neuronal structure and function^19^, yet their exact clinical efficacy requires further observation and confirmation. Laser therapy is capable of clearing drusen in AMD patients, but may cause inflammatory-related damage and is unable to prevent progression to advanced AMD^37,38^. Recently, scientists have performed clinical trials involving nanosecond laser treatment for RD, but long-term observation of its safety and effects is still needed^39^. An ideal RD-combating strategy that is generally safe, physiologically stable, highly cost-efficient and targeted at regrowth of retinal cells is appealing and urgently demanded. Fortunately, stem/progenitor cells, including retinal progenitor cells (RPCs), embryonic stem cells (ESCs), induced pluripotent stem cells (iPSCs) and mesenchymal stromal cells (MSCs), are capable of self-renewal and multidirectional differentiation, and have been extensively studied for biomedical applications to meet the ever-stringent requirements of clinical translation^40^. Numerous well-established preclinical studies of stem/progenitor cell-based therapies in various RD animal models have been conducted by replacing degenerative cells and/or providing nutritional support, and the results suggest great potential in the clinical treatment of RD. More recently, it has been discovered that many types of stem cells, e.g., adipose, bone marrow and umbilical MSCs, can secrete multifunctional exosomes that have low risk of toxicity and immunological rejection, and hold substantial potential for immunotherapy and drug delivery by transmitting numerous biomolecules to specific cells^41–44^. This implies that stem cells will gain additional traction as a promising treatment for RD due to the advantages of their secreted nanoscale exosomes^45^. A previous review has described in detail the present basic experiments and rationale behind stem/progenitor cell-based transplantation for treatment of RD^46^. Here, a series of clinical trials based on four stem/progenitor cells—RPCs, ESCs, iPSCs, and MSCs—for RD treatment will be comprehensively reviewed (Table 1), and the underlying working mechanism and the respective advantages and disadvantages of these cells will be discussed in the context of their future clinical application (Scheme 1).

**Table 1:** Stem/progenitor cell-based clinical trials for RD treatment.

Cell therapy	Identifier	Phase	Institution	Location	Study start date	Disease enrolment	Single dose
RPC	NCT02320812	I/IIa	jCyte	California, US	June 2015	28 RP	500,000–3,000,000 cells
	NCT03073733	IIb	jCyte	California, US	March 2017	82 RP	3,000,000 or 6,000,000 cells
	NCT02464436	I/II	ReNeuron Limited	Boston and Phoenix, US	December 2015	21 RP	No data
	ChiCTR-TNRC-08000193	I	Chongqing Science and Technology Commission	Chongqing, China	May 2008	8 RP	1,000,000 cells
ESC–RPE	NCT01345006 NCT01344993	I/II	Advanced Cell Technologies	California, US	April 2011	9 SMD 9 AMD (dry)	50,000–150,000 cells
	NCT01469832	I/II	Astellas Institute for Regenerative Medicine	London, UK	November 2011	12 SMD	50,000–200,000 cells
	NCT01625559 NCT01674829	I I/IIa	CHABiotech	Seoul, South Korea	September 2012	2 SMD 2 AMD (dry)	50,000 cells
	NCT01691261	I	Moorfields Eye Hospital NHS Foundation Trust	London, UK	June 2015	2 AMD (wet)	6.0 × 3.0-mm RPE sheet
	NCT02286089	I/II	CellCure Neuroscience	Jerusalem, Israel	April 2015	15 AMD (dry)	50,000–500,000 cells
	NCT02755428	I/II	Chinese Academy of Sciences	Beijing, China	January 2018	10 AMD (dry)	No data
iPSC-RPE	UMIN000011929	I	Riken	Kobe, Japan	August 2013	1 AMD (wet)	1.3 × 3.0-mm RPE sheet
	NCT02464956	/	Moorfields Eye Hospital NHS Foundation Trust	London, UK	July 2015	10 AMD (dry)	No data
BM-MSC	NCT01068561	I	University of Sao Paulo	Sao Paulo, Brazil	May 2009	3 RP 2 cone-rod dystrophy	10,000,000 cells
	NCT01560715	II			January 2011	20 RP	
	NCT02016508	I/II	Al-Azhar University	Egypt	March 2013	1 AMD (dry)	No data
BM CD34+ cells	NCT01736059	I	University of California, Davis	California, US	July 2012	6 RD or ischaemic disorders	3,400,000 cells
MSC-Exo	NCT03264976	/	Shanghai General Hospital, Shanghai Jiao Tong University School of Medicine	Shanghai, China	July 2018	200DR	No data

## Initial clinical trials based on stem/progenitor cells

After promising results from studies with a variety of animal models of RD, several initial clinical trials based on RPE or photoreceptors using human tissues or cells were initiated. The pioneering transplantation of foetal human RPE cells into the subretinal space of AMD patients without immunosuppression was initiated by Algvere et al.^47^. Host–graft rejection was observed in the exudative lesions over 1–6 months, while little evidence of rejection was detected after 12 months in geographic atrophy of dry AMD, suggesting a possible risk of rejection response in RPE cell transplantation. To further determine the long-term safety of hRPE transplantation in AMD patients, Algvere et al. then carried out another clinical trial by enrolling 16 patients with dry or wet AMD followed by a 2-year observation^48^. RPE-derived small extrafoveal transplants in the subretinal space of dry AMD patients without immunosuppression did not induce a rejection response, suggesting the potential biosafety of hRPE transplantation in an extrafoveal form for dry AMD treatment. Nevertheless, the improvement in visual acuity demands further confirmation. Almost at the same time, another clinical trial was conducted to establish the biosafety of photoreceptor cell implantation in RP patients. Kaplan et al. transplanted a sheet of photoreceptor cells into the subretinal space of two advanced RP patients without immunosuppression^49^. Twelve months after administration, their visual acuity remained the same (no light perception), and there was no evidence of immune rejection or clinical evidence of detrimental effects, which supports satisfactory biosafety; the effectiveness of cell-based transplantation remains to be exanimated. To further investigate the efficacy of neural retinal transplantation, Humayun et al. conducted a pilot study involving eight advanced RP patients who received human foetal retinal microaggregate suspension and one wet AMD patient who received both human foetal retinal microaggregate suspension and an undissociated retinal sheet^50^. During the first month, three RP patients exhibited transiently improved light sensitivity; however, the effect was reversed in the following months. Although no improvement in light sensitivity was ultimately observed, the study supported the potential effectiveness of cell-based transplanted strategies to some degree. Radtke et al. conducted a similar clinical trial to demonstrate the efficacy of retinal transplantation^51^. Foetal retinal sheets were transplanted subretinally in two RP patients, and threshold objective improvement of visual acuity was observed in one patient 4 months after the surgery. Subsequently, they co-transplanted sheets of foetal neural retina and RPE into the subretinal space of five advanced RP patients, in whom no vision enhancement was observed^52^. Fortunately, seven of ten patients (six RP patients and four AMD patients) experienced favourable outcomes of improved visual acuity in a follow-up clinical trial^53^, providing clinical evidence of the efficacy of cell-based implantation for RD therapy. Although the limited efficacy observed in these early clinical trials based on RPE cells and foetal retinal tissue transplantation did not meet the rigorous demand of commercial clinical translation for RD patients, the technical feasibility of cell-based implantation was preliminarily confirmed so as to support subsequent clinical trials with stem/progenitor cell transplantation.

## Stem/progenitor cell-based clinical trials for RD treatment

### RPCs

The mature mammalian retina was considered to lack regenerative capacity until the ciliary epithelium was discovered to be the retinal stem cell niche^54^. However, the limited proliferative capacity of pigmented ciliary margin cells in humans impedes their potential use in treating RD. Alternatively, RPCs are a type of neural progenitor cell (NPC) located in the inner layer of the optic cup^55^. Experiments have shown that in rodents, foetal and postnatal-derived RPCs express several developmental makers (e.g., nestin, Pax6, vimentin, Sox2, Ki-67, β-III tubulin and doublecortin^56–58^), suggesting that they possess proliferative capacity similar to that of other stem cells. More importantly, RPCs isolated from various gestational or postnatal periods of rat models can differentiate into various retinal cell types (e.g., bipolar neurons, rod photoreceptors and Müller glial cells^59,60^). The specific stem cell properties including proliferation and differentiation displayed by RPCs make RPC transplantation a promising avenue for RD treatment.

#### Progress of RPC-based clinical trials

**Fig. 1:**
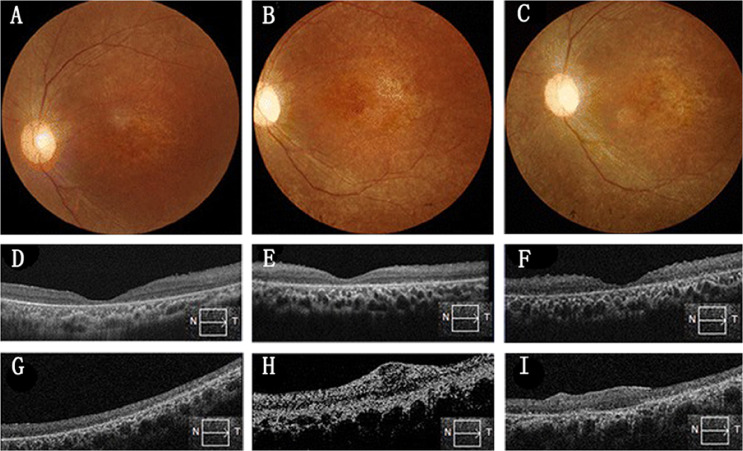
Colour fundus photographs and OCT images before and after retinal progenitor cell (RPC) transplantation for RP patients. **a**–**c** Colour fundus photographs at baseline (**a**), 12 months after transplantation (**b**) and 24 months after transplantation (**c**). No retinal haemorrhage of oedema was observed after transplantation. **d**–**f** Foveal OCT images at baseline (**d**), 12 months after transplantation (**e**) and 24 months after transplantation (**f**). No macular oedema was observed after transplantation. **g**–**i** Horizontal OCT image of the injection site at baseline (**g**) and after transplantation (**h** and **i**). Retinal scarring was observed in this patient (**h**, **i**). Injected RPCs disappeared 24 months after transplantation. Reproduced with permission^66^. Creative Commons Attribution 4.0 International License.

The favourable outcomes in previous fundamental research demonstrated that immature post-mitotic rod precursors used as donor cells can differentiate into rod photoreceptors and integrate into the degenerating retina, thereby improving visual function^61^. On the basis of these observations, scientists further tried to separate foetal tissue-derived RPCs (fRPCs) from the human retina between 14 and 20 weeks of gestation at the time when photoreceptor progenitors are differentiating, and found that these fRPCs can be donor cells for RD treatment^62^. In June 2015, the first FDA-approved Phase I/IIa clinical trial using fRPCs was initiated by Klassen et al. (NCT02320812)^63^. The study enrolled a total of 28 patients with RP, and various doses (0.5–3 million) of foetal tissue-derived RPCs were injected into their vitreous cavity as a cell suspension. Twelve months after transplantation, treatment-emergent adverse events (TEAE) were reported in 21 patients, including one patient who suffered from a grade-3 TEAE; improvement of mean best corrected visual acuity (BCVA) in the test eyes varied from 8 to 14 letters without much clinical significance. These outcomes demonstrated the acceptable safety and tolerability of hRPC transplantation that still needs to be improved. A subsequent Phase IIb study designed to evaluate changes in visual function of RP patients following a single injection of hRPCs has completed enrolment (NCT03073733)^64^. Similarly, another FDA-approved Phase I/II clinical trial (NCT02464436) enrolling 21 RP subjects was conducted at two institutes in Boston and Phoenix^65^. It is a dose-escalation study in which participants with RP received a single subretinal injection of hRPC cells in one eye to evaluate the safety, tolerability and effectiveness of the treatment. This study is still ongoing and is estimated to be completed in July 2021. Aiming to demonstrate the therapeutic effects of RPC-based transplantation, evaluating long-term safety and efficacy is a primary objective of this study. Fortunately, in Asia, Liu et al. evaluated the feasibility and long-term safety of hRPC transplantation in eight advanced RP patients through subretinal injection (ChiCTR-TNRC-08000193) (Fig. 1)^66^. Despite signs of retinal scarring observed after transplantation (Fig. 1h, i), no immunological rejection or tumorigenesis was observed during the 24-month follow-up, which indicated the long-term safety of hRPC transplantation. In addition to improved safety, a significant improvement of BCVA in five eyes and an increase of retinal sensitivity of pupillary response in three patients were also observed between 2 and 6 months after transplantation. However, the improvement did not continue through 12 months. Since the confirmed biosafety and feasibility of hRPC transplantation has laid a solid foundation for vision repair by RPC-based transplantation therapy, the next step is to improve the long-term efficacy of RPC transplantation in RD patients.

#### Main advantages and disadvantages of RPCs

The safety and effectiveness of RPC-based transplantation have been widely studied through a series of preclinical and clinical trials. Compared with other stem cells, the main issues facing RPC transplantation are the shortage of sufficient donor cells due to the limited proliferative capacity of RPCs^67^ and their restricted ability to differentiate into the specific targeted cells^68^. To address these issues, our group found that insulin-like growth factor-1 binds to its receptor and stimulates the PI3K/Akt and MAPK/Erk pathways through increased phosphorylation, resulting in accelerated RPC proliferation^69^. More recently, we synthesised a mussel-inspired injectable hydrogel and its counterpart that are capable of directing RPCs to differentiate towards retinal neurons and promoting proliferation of RPCs^70^. These studies suggest that the shortage of RPCs and limited targeted-differentiation capacity may be resolved. Together with the outstanding advantages of RPCs, such as avoiding ethical issues and the relatively low risk of immune rejection and tumorigenesis, RPCs are considered a good source of donor cells for further clinical RD treatment.

### ESCs

ESCs exhibit limitless proliferation and multi-differentiation into various cell types^71^. The neural progenitors derived from mouse ESCs express regulatory factors to induce retinal differentiation^72^, indicating that ESCs are able to differentiate into the photoreceptor lineage under certain circumstances in vitro and may potentially be an unlimited source for RD treatment.

#### Progress of ESC-based clinical trials

**Fig. 2:**
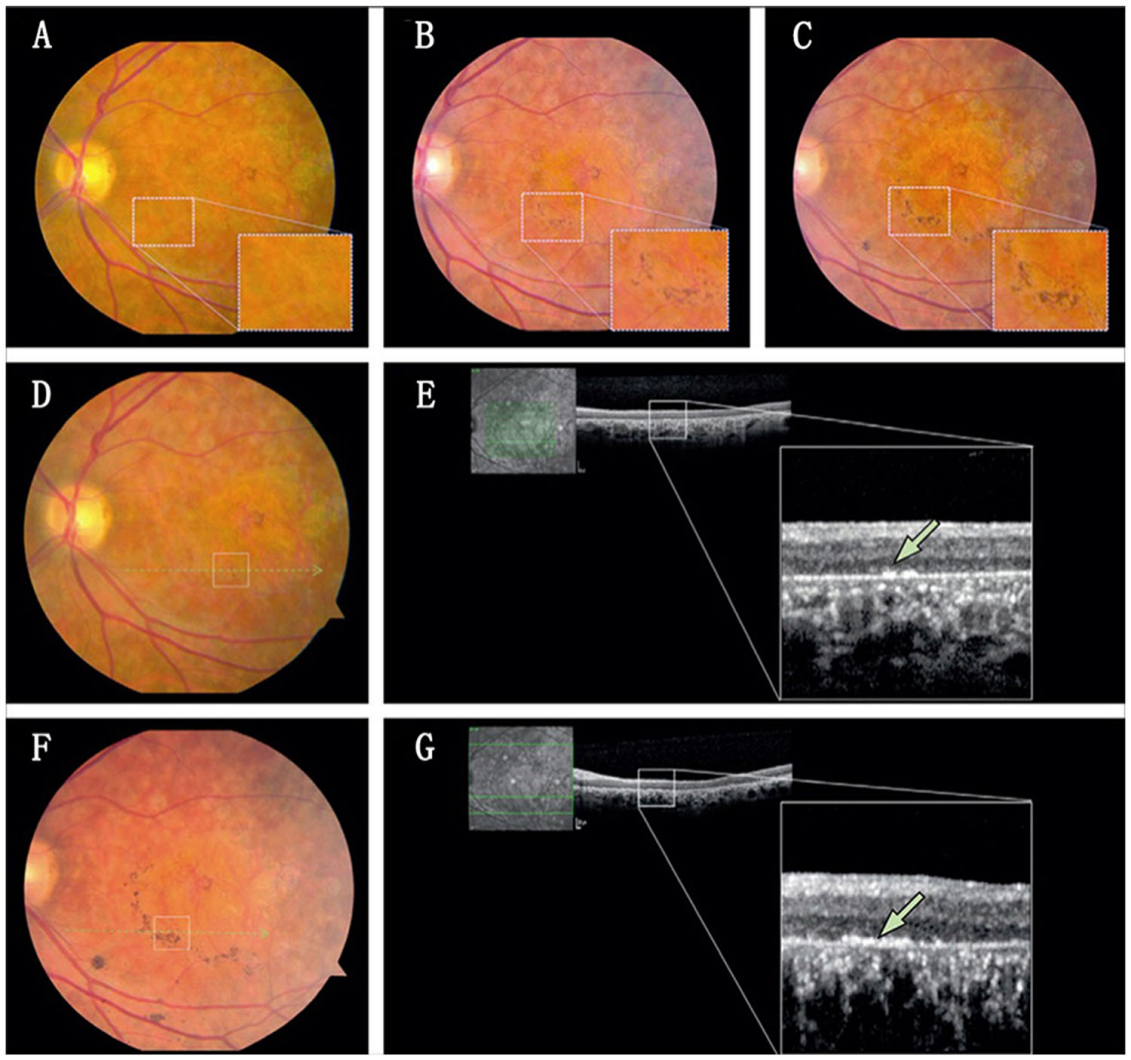
Colour fundus photographs and OCT images of the left macular in Stargardt’s macular dystrophy (SMD) patients before and after hESC-RPE transplantation. **a**–**c** Colour fundus photographs at baseline (**a**), 1 week after transplantation (**b**) and 6 weeks after transplantation (**c**). **d**, **f** Macular colour images at baseline (**d**) and 3 months after transplantation (**f**). **e**, **g** OCT images at baseline (**e**) and 3 months after transplantation (**g**). Colour fundus photographs showed that pigmentation increased continuously from baseline to months 3, and OCT images showed that it is at the level of RPE. Reproduced with permission^74^. Copyright 2012 Elsevier Ltd.

Preclinical animal models have shown that ESCs can differentiate into a range of retinal cell types, and several clinical trials demonstrated the efficacy and safety of ESC transplantation for the treatment of RD^73^. In April 2011, Schwartz et al. received the first authorisation from the FDA to initiate a Phase I/II trial involving subretinal transplantation of a low dose of hESC-RPEs (NCT01345006 and NCT01344993) (Fig. 2)^74^. The study enrolled one participant with Stargardt’s macular dystrophy (SMD) and one with dry AMD. Four months after transplantation, visual acuity improved from 0 to 5 in the SMD patient and from 21 to 28 in the dry AMD patient, suggesting effectiveness of ESC-based transplantation therapy. It is also encouraging that no adverse proliferation or rejection was detected during the observation period. Since the safety of hESC-RPE transplantation had been demonstrated, in subsequent studies, three dose cohorts (50,000, 100,000 and 150,000 cells) were implanted into nine SMD participants and nine dry AMD participants^75^. The BCVA in the ten treated eyes and vision-related quality-of-life measures for general and peripheral vision improved 22 months after transplantation with no evidence of serious systemic or ocular adverse reactions. These results supported that hESC-RPEs can be donor cells for RD treatment. Encouraged by these outcomes, in November 2011, a more comprehensive investigation of hRPE-ESC transplantation with a higher dose of hESC-RPE cells was conducted in the United Kingdom (NCT01469832)^76^. The investigation enrolled 12 patients suffering from advanced SMD with systemic immunosuppression who received transplantation with one of four doses of hESC-RPE cells (50,000, 100,000, 150,000 or 200,000 cells) into the subretinal area. No evidence of uncontrolled proliferation or inflammatory responses was found even after subretinal administration of up to 200,000 cells; however, evidence of benefit at 12 months was also not observed. Although ESC-based transplantation therapy can theoretically improve visual acuity in 12 patients, it was not clinically meaningful due to the severity of retinal degeneration. Even though the ESC transplantation has been conducted for over three decades and has some promising results, there have been no reports on the safety and effectiveness in Asian RD patients. To expand the universality of this treatment, in September 2012, Song et al. initiated the first clinical trial in Asia involving four RD patients (two SMD patients and two dry AMD patients) (NCT01625559 and NCT01674829) by injecting a low a dose of hESC-RPEs (50,000 cells) per eye^77^. After 12 months of follow-up, three patients exhibited 9–19 letter improvement of visual acuity, which showed that transplantation of hESC-RPE to treat RD is also effective in Asian patients. Since advanced RD ultimately leads to blindness, scientists concentrate on how to improve the visual acuity of these patients. In June 2015, Lyndon et al. performed a Phase I clinical trial with two severe wet AMD patients by delivering an RPE patch containing a hESC-derived RPE monolayer and a basement membrane into the subretinal space (NCT01691261)^78^. One patient had a 29-letter improvement in visual acuity and one improved by 21 letters after 12 months, which supports the feasibility of RPE patch transplantation in advanced RD treatment. The RPE patch may be a better alternative than cell suspensions. More recently, patients with RD in some clinical trials are being prepared. In Jerusalem (Israel), a Phase I/II clinical trial using OpRegen (a cell-based product composed of RPE cells derived from hESCs) to treat dry AMD (NCT02286089) is still recruiting and is estimated to be completed in December 2024^79^. Similarly, in Beijing (China), a Phase I/II clinical trial of subretinal transplantation of hESC-RPEs (NCT02755428) is currently enrolling patients with dry AMD and is estimated to be completed in December 2020^80^. In summary, ESCs display an enormous potential for RD by providing millions of target cells required for transplantation.

#### Main advantages and disadvantages of ESCs

Compared with harvesting RPCs, it is relatively easy to obtain sufficient ESCs for transplantation. Nevertheless, ESCs have potential for tumour formation due to their high proliferative capacity. Chaudhry et al. integrated both ESCs and ESC-derived neural progenitors into the diseased retinal tissue of rd12 mice, and the proliferation of ESCs eventually resulted in teratoma formation, while ESC-derived neuroprogenitors integrated into the retinal layers^81^. This outcome not only demonstrated the possible tumorigenicity of ESC transplantation, but highlighted that differentiation of ESCs into neuroprogenitors before transplantation may decrease the risk of tumour formation. Accordingly, the existing clinical trials always transplant ESC-derived RPEs into RD patients to reduce the possibility of tumorigenicity. However, ESCs isolated from foetal tissues may be surrounded by ethical concerns^73^, and the multidirectional differentiation also presents difficulties in obtaining the targeted cell types^82^. Moreover, the requirement for lifelong immunosuppressive therapies presents risk and economic burden that further threaten the potential of ESC transplantation^83^. In summary, there are still many challenges to overcome before the clinical use of ESC-based transplantation for RD therapy.

### iPSCs

In 2006, Takahashi and Yamanaka introduced four factors—Oct3/4, Sox2, c-Myc and Klf4—into mouse embryonic fibroblasts under ES cell culture conditions to induce the iPSC state^84^. The iPSCs were capable of differentiating into all three germ layers after transplantation into foetal and adult mice. One year after the iPSC discovery, they reprogrammed differentiated human somatic cells into a pluripotent state with the same four factors^85^. After that, the safety and efficiency of iPSC generation was improved over the last decade. Scientists found that using factors such as valproic acid^86^, SV40 large T antigen^87^ and microRNA^88^ can improve the efficiency of pluripotent induction. Furthermore, the iPSC production safety problem was solved by Nakagawa et al. without the use of Myc^89^. Since the differentiation potentials of iPSCs and ESCs are similar, scientists have also employed iPSC-derived cells to treat RD.

#### Progress of iPSC-based clinical trials

**Fig. 3:**
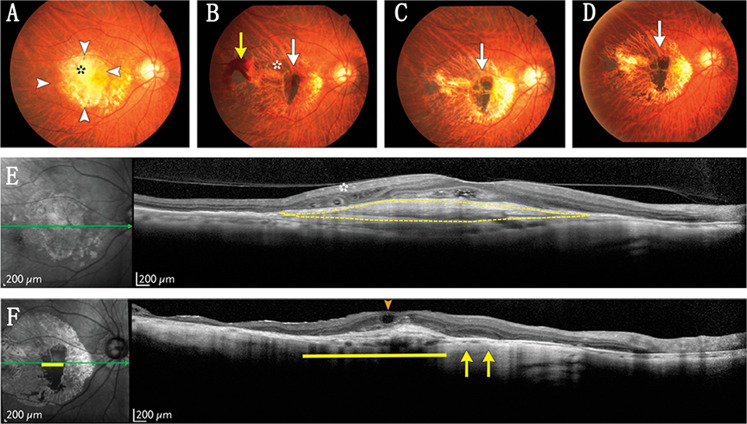
Colour fundus photographs and OCT images of the right macular in the wet age-related macular degeneration (AMD) patient before and after induced pluripotent stem cell (iPSC)-RPE transplantation. Preoperative and postoperative colour fundus photographs (**a**–**d**) and OCT images (**e**, **f**) of the iPSC-derived RPE sheet transplantation site in the AMD patient. There were a fibrotic neovascular membrane and polys before transplantation (**a**). The RPE sheet was curled on day 3 after the surgery (**b**), but flattened after 8 weeks (**c**) and lasted until 1 year after transplantation (**d**). One year after the surgery, the graft sheet could still be observed. OCT images showed that there was a large hyperreflective mass before treatment (**e**), but it disappeared at postoperative 1 year (**f**). Reproduced with permission^90^. Copyright 2017 Massachusetts Medical Society.

The first clinical trial using iPSC-RPE subretinal transplantation was initiated by Takahashi’s group (RIKEN in Kobe, Japan) in August 2013 (UMIN000011929)^90^. The study enrolled a 77-year-old Japanese female, who became the first person in the world to receive an autologous iPSC-derived RPE sheet implantation (Fig. 3). One year after transplantation, her vision reduction was stabilised without adverse effects^91^. However, the trial was forced to stop in the subsequent year because of mutations in the second patient’s iPSCs and regulatory changes in Japan. Although the investigators declared that mutations are not necessarily tumorigenic, safety issues still need to be reconsidered in human trials, as other scientists have documented genomic instability in iPSCs^92^. To continue the study, Takahashi et al. investigated HLA-matched allogeneic iPSC-derived RPE cells^93^. On 28 March 2017, they enrolled the first 60-year-old Japanese male to receive allogenic iPSC-RPEs in suspension. Compared with autologous iPSCs, the HLA-matched allogeneic iPSCs were safer to administer and more likely to succeed financially. More recently, in Moorfields Eye Hospital in England, an FDA-approved clinical trial of iPSC-RPE subretinal transplantation enrolling ten dry AMD patients is ongoing (NCT02464956)^94^. In the United States, groups of scientists are seeking FDA approval for their clinical studies^95^. Many scientists have aimed to address the safety concerns surrounding iPSC transplantation through the reprogramming process^96^; however, it remains to be seen whether the modifications will work.

#### Main advantages and disadvantages of iPSCs

As mentioned above, iPSCs ameliorate the ethical issues of ESCs and have the potential for reduced immunogenicity through autologous transplantation, but iPSCs have a lower variable differentiation efficiency and a relatively high risk of gene mutation^97^. Thus, iPSCs are expected to replace ESC-based therapy in RD treatment.

### MSCs

As a therapeutic option for RD, MSCs mainly provide trophic support via a paracrine mechanism to slow retinal degeneration instead of replacing damaged cells^98^. BM-MSCs and ADSCs are two main sources of MSCs with the ability to differentiate into neural retinal cells. Since the feasibility of intravitreal autologous BM-MSC transplantation was demonstrated by Jonas et al.^6^, various clinical trials of BM-MSC transplantation have been carried out^99^. Studies have demonstrated that BM-MSCs and ADSCs share similar immunomodulatory capacities^100^. Therefore, ADSCs are considered an alternative to BM-MSCs for RD treatment, despite the few current studies on ADSC-based therapy.

#### Progress of MSC-based clinical trials

**Fig. 4:**
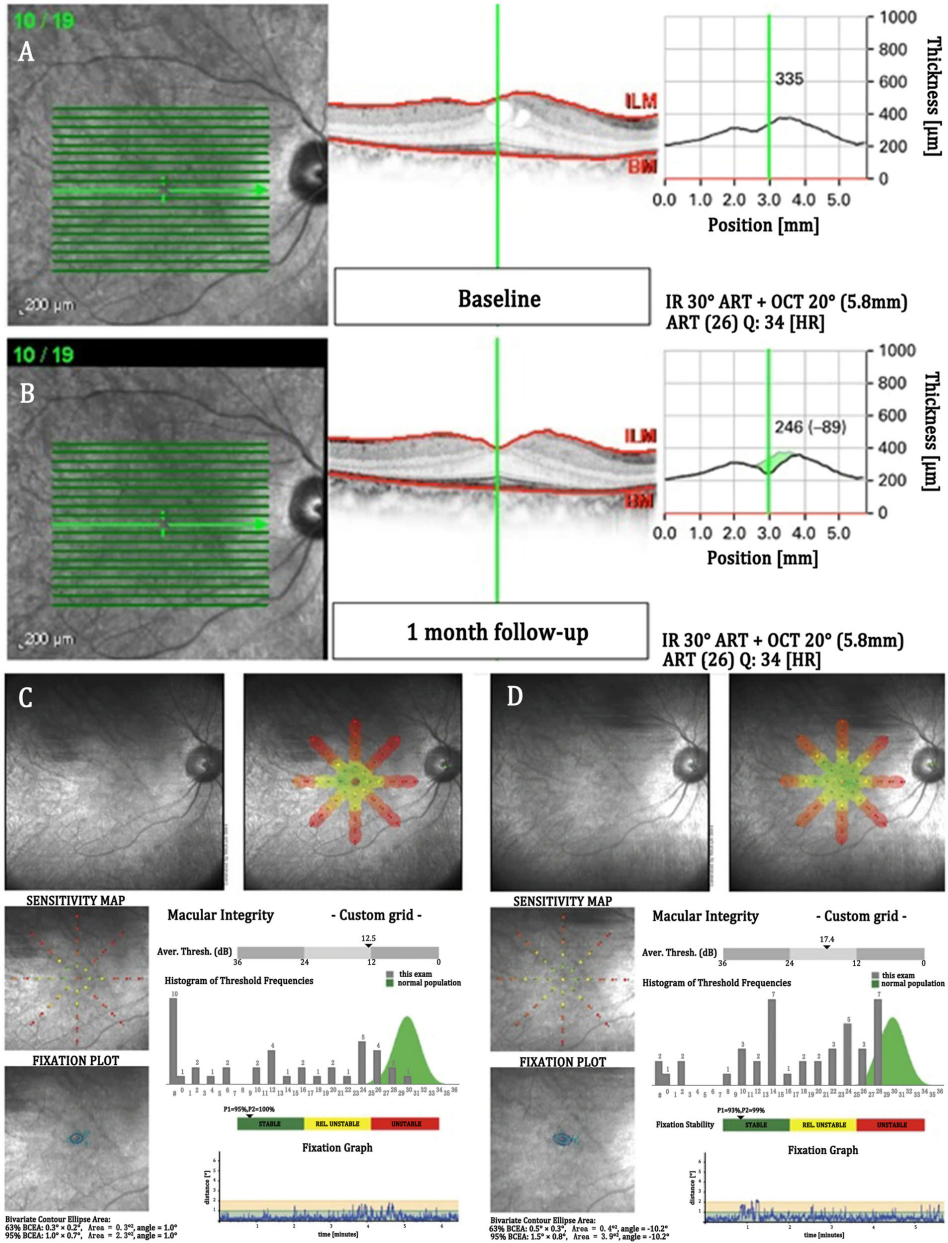
OCT and microperimetry examinations of RP patient with cystoid macular oedema before and after BM-MSC transplantation. **a**, **b** Macular thickness of the patient at baseline (**a**) and 1 month after the transplantation (**b**). OCT examinations showed that macular oedema was eliminated after 1-month follow-up. **c**, **d** Macular sensitivity of the patient at baseline (**c**) and 1 month after the transplantation (**d**). Microperimetry showed that macular sensitivity increased after 1-month follow-up. Reproduced with permission^102^. Copyright 2012 Springer Nature.

The progress of BM-MSC transplantation therapy has been accelerated towards the clinic. In May 2009, Siqueira et al. initiated a Phase I trial in three patients with RP and two patients with cone-rod dystrophy by injecting autologous BM-MSCs into the vitreous cavity (NCT01068561) (Fig. 4)^101,102^. Ten months after transplantation, there was no detectable structural or functional toxicity, demonstrating the short-term safety of the transplantation. Based on the promising result, a Phase II study was initiated in June 2011 to further confirm the efficacy of BM-based transplantation therapy (NCT01560715)^103^. The study enrolled 20 RD patients who received a vitreous implantation of BM-MSCs, finding that vision-related life quality improved 3 months post treatment, supporting the potential efficacy of BM-MSC therapy; however, it was transitory and no longer evident at 12 months post treatment. Similarly, 1 year later, another pilot clinical study was initiated by Park et al. by injecting autologous CD34^+^ BM-MSCs into the vitreous cavity of six patients with retinal vascular occlusion or RD (NCT01736059)^104^. The study is ongoing, and preliminary findings from the Phase I patients (six patients enrolled between November 2012 and August 2014) have been published. Within 6 months of follow-up, the autologous cells appear to be well tolerated, yet efficacy still requires further exploration. These unsatisfactory results may be due to the mechanism of BM-based transplantation therapy, namely, the neurotrophic effect that can only support the survival of photoreceptors instead of promoting their regeneration. More recently, an ongoing BM-MSC-based therapy for RD in Saudi Arabi was registered online (NCT02016508)^105^. There are increasingly safety concerns surrounding intravitreal administration of autologous BM-MSCs. Satarian et al. conducted a Phase I clinical trial to examine the safety of autologous BM-MSC transplantation. Three patients with advanced RP were enrolled and received intravitreal injection of autologous BM-MSCs^106^. After 2 years of follow-up, severe fibrous tissue proliferation was observed in the injection site of the third patient, leading to iris neovascularisation, formation of mature cataracts and tractional retinal detachment. Both the loss of improvement over time and the existing safety concerns may imply that BM-MSCs are not the best choice for RD treatment.

#### Main advantages and disadvantages of MSCs

To date, scientists have shown the neurotrophic effect of MSCs for treating RD^107^; however, little evidence of cellular replacement has been established. The cytokines^108^, growth factors^99,109^, micro- and nanolipid macrovesicles^110^ secreted by MSCs exert important angiogenic, immunomodulatory, anti-apoptotic and anti-inflammatory effects that provide trophic support for the degenerative retina. Further, the neurotropic effect cannot solve the intrinsic problem of RD, which is the fatal flaw of MSC-based transplantation therapy. In addition, adverse effects, e.g., iris neovascularisation observed in the eye receiving autologous MSC cell transplantation for RD therapy, may be partially caused by the lack of quality control of MSCs injected as autologous transplants and the high variability in MSCs derived from different individuals^106^, thus raising safety concerns of MSC-based transplantation therapy for RD. Moreover, the potential heterogeneous nature of MSCs restricts them from expanding into specific cells such as BM CD34+ cells, which may also be an issue for MSCs^111^. Last but not least, MSCs primarily differentiate into mesodermal-derived tissues far from the targeted retinal cells, and their regenerative potential declines with increasing donor age^112^. This further limits their promise as an RD treatment even if MSCs demonstrate immunosuppressive effects and are less immunogenic than other stem cells^113,114^.

## Stem cell-derived exosomes for RD treatment

In addition to replacement and neurotrophic mechanisms based on stem/progenitor cells, the latest studies have focused on extracellular exosomes secreted by stem cells as a potential therapy for RD. Several studies have identified that some types of MSCs secrete exosomes (MSC-Exo), mainly adipose, bone marrow and umbilical MSCs^45^. Exosomes are cell-derived nanovesicles that have low toxicity, low risk of immunological rejection, exquisite target-homing specificity and potential for drug/gene delivery^115^. Accordingly, exosome-based therapy is gaining research attention for multiple diseases throughout the body^45^. For instance, scientists have found that MSC-Exo has a prominent therapeutic effect in central nervous system diseases, such as stroke^116,117^, Alzheimer’s disease^118^ and spinal cord injury^119^. Importantly, the positive roles of MSC-Exo in anatomical and functional restoration of ocular tissues, e.g., cornea, optic nerve and retina, have also been confirmed in several types of eye diseases, including optic nerve crush^42^, glaucoma^120^, retinal ischaemia^121^ and DR^122^, by modulating angiogenesis and inflammation pathways, immunomodulation or even tissue regeneration. As reported, Safwat et al. transplanted rabbit adipose MSC-Exo to the eyes of rabbits with diabetes mellitus (DM) by subconjunctival and intravitreal injection. They found that the implanted MSC-Exo efficiently delivered miRNA-222 into the retina to prevent the progression of retinal degeneration^122^. Similarly, Zhang et al. discovered that human umbilical cord MSC-Exo reduces hyperglycaemia-induced retinal inflammation after intravitreal injection into rabbis with DM^123^. Based on the high efficacy of MSC-Exo for RD control in preclinical trials, a clinical trial intending to evaluate the function of serum exosomal miRNA in the pathogenesis of DR has obtained FDA approval (NCT03264976) and will soon recruit patients^124^. Stem cell-derived exosomes may play an important role in RD treatment in the future.

## Conclusions

RD is a leading cause of blindness worldwide, and it mainly results from the degeneration of RPE and photoreceptor cells. Accordingly, scientists have applied progenitor/stem cell-based transplantation therapy to RD treatment, especially the use of RPC-, ESC-, iPSC- and MSC-based therapies, by replacing degenerative retinal cells and/or preventing retinal degeneration by supplementing trophic factors. Most importantly, stem cells also secrete multifunctional exosomes and serve as pathogenic mediators between cells in the eyes, which are essential working mechanisms underlying how stem cell-based therapy impacts RD. Through thorough discussion on the advantages and disadvantages of different kinds of stem/progenitor cells, we hypothesise that RPCs are the best candidates for RD treatment since they do not present ethnical concerns and they have a relatively low risk of rejection and tumorigenesis. Although there are some limitations in RPC proliferation and differentiation using current technologies, emerging culture techniques (as described above) will provide an opportunity to solve the problems. In addition, to increase the safety and efficacy of stem/progenitor cell-based transplantation therapy, several unsolved issues and corresponding strategies need to be resolved:

1. Currently, research on stem cell-derived exosome-based strategies for biomedical applications is still in its infancy. One of the main weaknesses of nanovesicle exosomes is their rapid clearance from tissues or organs. Clearance should be verified in ophthalmology after topical, intravitreal or subconjunctival applications to determine how to overcome this issue. On the other hand, exosomes as a sustained delivery platform greatly rely on the generation of vesicles of consistently high purity and quality on a large scale, which is another challenge. Hence, more methods and technologies for exosome-based systems should be developed to generate the next stem cell-derived exosome nanomedicine for RD management.
2. The proliferation and differentiation mechanisms of stem/progenitor cells still require better understanding. Thus, besides clinical trials, more basic experiments need to be conducted for a deeper comprehension of the mechanism, which will facilitate the application of progenitor/stem cell-based transplantation therapy in more RD patients as early as possible.
3. Potential tumorigenicity of stem cells and immune rejection caused by exogenous transplantation strategies does not meet the clinical safety threshold. Fortunately, fundamental experiments have shown that pre-induction of ESCs into neural progenitors before transplantation may reduce tumorigenicity^81^, providing a feasible solution to this problem. Meanwhile, immunosuppressive therapies also evolve, and new techniques such as the xeno-free techniques are being developed to reduce the immune response. Thus, the issue of immune rejection is expected to be addressed in the future.
4. Ethical issues of stem cell transplantation need to be resolved. On one hand, iPSC generation does not present the same ethical concerns as ESC harvesting. On the other hand, iPSC-based strategy is a completely new field and remains in its infancy; experts will reach an ethical consensus over time.
5. Most of the current clinical trials are in the early I/IIa phases. Thus, there is still a long way to go before their findings can be applied to clinical practice.

All in all, with deepening research, stem/progenitor cell-based transplantation will be an essential treatment used in the clinic that will bring new hope to RD patients through the joint efforts of doctors and researchers.
